# Rasch analysis of the living with chronic illness scale in Parkinson’s disease

**DOI:** 10.1186/s12883-020-01926-7

**Published:** 2020-09-15

**Authors:** Leire Ambrosio, Carmen Rodriguez-Blazquez, Alba Ayala, Maria João Forjaz

**Affiliations:** 1 Faculty of Nursing, University of Navarre, Campus Universitario s/n, 31009 Pamplona, Navarre Spain; 2 National Centre of Epidemiology, Institute of Health Carlos III and CIBERNED, Madrid, Spain; 3 National School of Public Health, Institute of Health Carlos III and REDISSEC, Madrid, Spain

**Keywords:** Parkinson’s disease, Living with, Rasch analysis, Psychometrics, LW-CI-PD

## Abstract

**Background:**

Neurologists play an essential role in facilitating the patient’s process of living with Parkinson’s disease (PD). The Living with Chronic Illness Scale-PD (LW-CI-PD) is a unique available clinical tool that evaluates how the patient is living with PD. The objective of the study was to analyse the LW-CI-PD properties according to the Rasch model.

**Methods:**

An open, international, cross-sectional study was carried out in 324 patients with Parkinson’s disease from four Latin American countries and Spain. Psychometric properties of the LW-CI-PD were tested using Rasch analysis: fit to the Rasch model, item local independency, unidimensionality, reliability, and differential item functioning by age and gender.

**Results:**

Original LW-CI-PD do not fit Rasch model. Modifications emerged included simplifying the response scale and deleting misfit items, the dimensions Acceptance, Coping and Integration showed a satisfactory fit to the Rasch model, with reliability indices greater than 0.70. The dimensions Self-management and Adjustment to the disease did not reach fit to the Rasch model.

**Conclusion:**

Suggestions for improving the LW-CI-PD include a multidimensional and shorter scale with 12 items grouped in three subscales with a simpler response scheme. The final LW-CI-PD Scale version is a reliable scale, with good internal construct validity, that provides Rasch transformed results on linear metric scale.

## Background

Parkinson’s disease (PD) is the second most common neurodegenerative chronic condition with a prevalence of 1% in people over the age of 60 affecting 10 million people worldwide [1]. It is a complex and disabling disorder manifested through a combination of motor and non-motor symptoms that generate an important impact on the daily living [2]. Throughout the PD course, patients do not only experience a progressive intensification of symptoms, but also have to deal and cope with an increasing limitation in all areas of their day to day, as a consequence of the disease [3, 4]. Several studies show that living with PD affects the patients’ physical state together with other essential aspects in their lives, such as the psychological, social and spiritual ones [5, 6]. Consequently, evaluating how patients live with PD using clinical measures becomes fundamental for the healthcare system [7].

The Living with Chronic Illness Scale-Parkinson’s disease (LW-CI-PD) scale is the only available instrument in clinical practice and research to evaluate the degree of living with a neurodegenerative condition as PD from the patient perspective [8]. The LW-CI-PD is composed by the following five sections: domain 1. Acceptance refers to recognize and assume the disease (4 items); domain 2. Coping alludes to face with PD (7 items); domain 3. Self-management refers to know what to do and how to control the disease (4 items); domain 4. Integration means making the disease part of the day-to-day of the person (5 items); and domain 5. Adjustment refers to the transformation that the person living with PD suffers not only in the daily living but also in his/her self-identity (6 items). The LW-CI-PD is a new self-reported measuring scale that has been recently validated in PD and other prototypical chronic conditions showing satisfactory psychometric properties [8, 9]. All studies have been carried out by using a classic test theory approach to evaluate reliability, validity and sensitivity to change along with acceptability and other parameters, mostly based on correlations and mean-difference analyses. The application of the Rasch measurement analysis [10] combined with the classic test theory approach is recommended for evaluating patient outcome report measures [11].

The Rasch model [10] is one of the most used applications of item response theory, completing the information provided by the classic test theory because it provides additional and relevant information about the measurement properties of a scale, like for example the LW-CI-PD. The Rasch model assess the internal construct validity, the optimal scoring scheme, unidimensionality, item fit, item local independency, and item bias by subgroups of respondents. Scales that fit the Rasch model provide interval linear measure that establishes equal intervals between the values and could be used in parametric analysis.

The objective of the present study was to analyze the LW-CI-PD properties according to the Rasch model. Suggestions for modification of the LW-CI-PD are presented accordingly.

## Methods

### Design

International and cross-sectional study of a sample of consecutive patients living with PD. This study is part of a bigger research project with the general aim to achieve a unique and international self-reported scale to evaluate the process of Living with PD as other long-term conditions all over the word (Spain, South America, UK).

### Participants

A consecutive cases sampling was applied to participant identification. The sample was formed by 324 patients with PD attended in specialized units of movement disorders of seven public and private centres from Cuba (*n* = 50), Argentina (*n* = 60), Ecuador (*n* = 60), Mexico (*n* = 53) and Spain (*n* = 101). Inclusion criteria were: 1) patients with a PD diagnosis by a neurologist according to international criteria [12]; 2) native Spanish-speaking patients; 3) able to read and understand properly questionnaires; and 4) able to provide informed consent. Exclusion criteria were: 1) patients with other neurodegenerative disorders related to PD; 2) cognitive deterioration previously and formally diagnosed by the neurologist; 3) acute disorder or other pharmacological effects that negatively influenced in the assessment; and 4) refusal to participate in the study.

### Assessments

Sociodemographic data such as age, gender, marital status, employment situation and educational level were collected. Also, PD historic data were collected, such as age at PD onset, PD duration and PD treatment duration. All scales were used in the respective Spanish versions. In addition to sociodemographic and PD historic data, the following measures were used by the neurologist:

- Hoehn and Yahr (HY) stage [13] is a worldwide used tool to measure the overall severity of PD, which comprises five stages.
- Scales for Outcomes in Parkinson’s Disease-Motor (SCOPA-M) [14] is a specific PD measure to evaluate motor symptoms. Its 21 items are grouped into the following three sections: 1) examination, 2) daily living activities and, 3) motor complications. The score for each item ranges between 0 (normal, no affectation) and 3 (severe affectation) and the total scale score is 0–75.
- Clinical Impression of Severity Index-PD (CISI-PD) [15] it is a scale to evaluate the global impression of PD severity of the patient according to the following four areas: 1) motor signs, 2) disability, 3) motor complications, and 4) cognitive status. The total score ranges from 0 to 24.
- LW-CI-PD was also used [8]. It is a 26-item self-reported scale that measures the degree of living with PD in terms of acceptance (4 items), coping (7 items), self-management (4 items), integration (5 items) and adjustment (6 items) [8]. It is scored on a 5-point Likert-type scale ranging from 0 (never/nothing) to 4 (always/a lot). LW-CI-PD total score ranges from 0 (negative living with PD) to 104 (positive living with PD).

### Data collection

Participant recruitment process was carried out between May 2018 and June 2019. The participant of this study that was people living with PD filled in the scales during the consult with their neurologist. In order to ensure homogeneity and reproducibility of the procedure of data collection a standardized protocol was established with clear steps as explaining the research study slowly; asking about doubts; reading out load instructions of the scales and its answer options; writing a check mark in the answer chosen by the patient and giving participants time to complete it. The median time to complete all the measures was approximately 30–40 min.

### Ethical aspects

The study was approved by the Ethics Committee of the University of Navarre (reference number 071/2014) and all included centres. Patients signed the written consent to participate voluntarily without any compensation for this, after receiving the appropriate information. All data, and information about the participants’ identity, were handled in full confidentiality throughout the research process.

### Data analysis

The Rasch model purports that an answer to an item is a function of the item’s difficulty (or level of measured construct) and the person’s ability (or experienced level by the person), expressed in logits. There are several excellent tutorials that explain in detail Rasch analysis for health sciences [16, 17], so here only the main aspects are summarized. The following measurement properties were assessed: fit to the Rasch model, unidimensionality [18], appropriateness of the response scale, item local independency, reliability (Personal Separation Index, PSI), and item-person distribution. Differential item functioning (DIF) was tested by age (split by the median: up to 68 years, 68 years and more) gender, country, PD duration (split by the median: up to 10 years, 10 years and more) and HY stage [19]. We used the top-down purification approach to see if DIF cancelled out [20]. Rasch analyses were performed iteratively, with small modifications of the scale until model fit was achieved. The partial credit model for polytomous items was used as indicated by the likelihood ratio test (*p* < 0.001) [21]. We used the statistical software RUMM2030 to perform the Rasch analyses [22].

## Results

Three hundred tw enty four patients with PD, 52.78% men, mean age (standard deviation, SD) of 66.67 (SD: 10.68; range: 36–94) years were included. Two thirds (66.36%) were married, 42.90% were retired, and 31.79% had primary or secondary education levels. The distribution according the HY stages was: 8.64% in stage 1; 55.25%, stage 2; 30.56%, stage 3; 4.01, stage 4; and 1.54% in stage 5. The SCOPA-M score for the total sample was 22.34 (SD: 11.26), and for the CISI-PD 7.61 (SD: 4.00).

Analyses were performed separately for each one of the dimensions. Initially, all dimensions showed a misfit to the Rasch model, and thus modifications were performed iteratively until a model fit was achieved. Nevertheless, dimensions Self-management and Adjustment to the disease did not reach model fit. Table 1 shows the fit indices of the Acceptance, Coping and Integration dimensions, after model modifications, to the Rasch model. Item estimates are presented in Table 2.

**Table 1:** Fit of the LW-CI-PD dimensions to the Rasch model (results for Acceptance, Coping and Integration dimensions are shown.after model modifications)

		Ideal values	Acceptance	Coping	Self-management	Integration	Adjustment to the disease
**Number of items**			3	6^a^	4	4	6
**Item residual**	**Mean**	0	0.175	0.366	0.283	0.238	0.795
	**SD**	1	1.011	1.248	1.358	1.203	1.149
**Person residual**	**Mean**	0	−0.209	−0.565	0.972	0.748	0.249
	**SD**	1	2.167	1.577	1.688	1.562	1.141
**Chi-square**	**Value**		17.296	33.471	29.21	28.232	43.206
	**Prob.**	> 0.05/number of items	0.134	0.015	0.0006	0.0297	0.000146
**PSI**		> 0.70	0.798	0.765	0.67	0.728	0.663
**Unidimensional test CI test Binomial**		(LCI < 0.05)	0.0401 (0.016, 0.064)	0.0216 (−0.002, 0.045)	0.0185 (−0.005, 0.042)	0.0401 (0.016–0.64)	0.0154 (− 0.008, 0.039)

*SD* Standard deviation, *PSI* Person separation index, *Prob*. probability, *CI* Confidence interval, *LCI* Lower confidence interval

^a^ Included two items 9, for younger and older adults Unidimensionality results before splitting item 9

**Table 2:** Individual item fit residual for final Rasch analysis of the Acceptance, Coping and Integration dimensions

Item (response categories)	Location	Standard Error	Fit Residual	Chi-Square (df = 4)	Probability
**Acceptance**					
**1.** I get upset every time I think I have PD (0–3)	− 0.459	0.104	0.972	6.775	0.148
**3.** I get angry when having PD symptoms (0–4)	0.238	0.081	−0.962	7.341	0.119
**4.** It bothers me to change the way I live and sacrifice important things in my life because of PD (0–4)	0.221	0.081	0.515	3.181	0.528
**Coping**					
**5.** I try to cope and fight the diesase	−0.220	0.081	0.289	3.274	0.35127
**6.** I am interested in looking for things that motivate me not to focus only in PD (0–3)	0.204	0.082	0.516	4.614	0.20237
**7.** I try to see the positive side of PD (0–3)	0.123	0.079	2.01	10.159	0.01726
**9.** (younger age). I hope the situation with PD improves (0–3)	−0.329	0.098	−0.524	8.202	0.04202
**9.** (older age). I hope the situation with PD improves (0–3)	0.124	0.087	−1.448	5.796	0.12197
**10.** I have someone who listens to me and understands what I am living with PD (0–3)	0.099	0.080	1.352	1.427	0.69931
**Integration**					
**16.** I have integrated PD in my daily living and all things related to it (0–3)	−0.148	0.084	1.786	5.225	0.265
**17.** Despite PD, I lead a life as normal as possible (0–3)	−0.240	0.085	−1.035	15.345	0.004
**19.** I am as interested and I have the same illusion in leisure activities as before having PD (0–3)	0.414	0.085	0.483	5.393	0.249
**20.** Although I have PD I feel satisfied with my life (0–4)	−0.026	0.073	−0.281	2.269	0.686

*PD* Parkinson’s disease, *Df* degrees of freedom

**Fig. 1:**
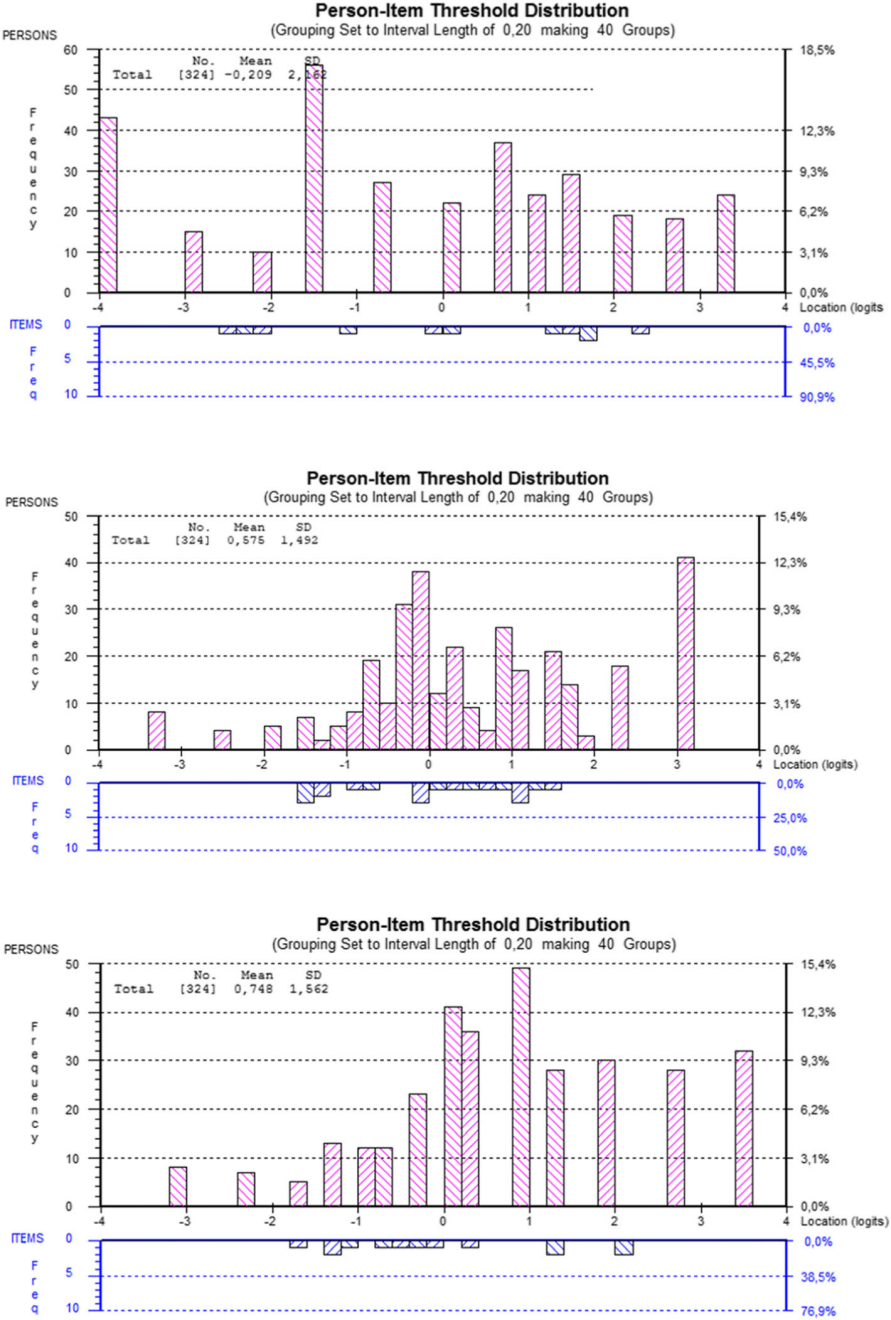
Person-item distribution for dimensions Acceptance (top), Coping (middle) and Integration (bottom) dimensions, after model modifications

The following modifications were performed to the dimension Acceptance: rescore item 1 (I get upset every time I think I have PD) by collapsing the second and third response categories, and deletion of item 2 (I am ashamed of PD and I hide it to others) due to a high fit residual (2.519). The resulting 3-item scale showed a good fit to the Rasch model, χ^2^(12) = 17.296, *p* = 0.138, PSI of 0.795, item local independency, unidimensionality, and absence of DIF by age, gender, PD duration and HY stage. However, DIF by country was found in all items, with an inconsistent pattern across items. The item distribution presented mean (M) = 0.175 logits, SD = 1.011, and a no floor effect (13.3%). Fig. 1 shows the person item-distribution.

Several item of the Coping dimension showed disordered thresholds, which were corrected by collapsing categories 2 and 3. In addition, items 8 (I try to lean on things that are important in my life), and 11 (I express my feelings so other people know how I feel and could help me) were deleted due to misfit (− 2.950 and 8.505, respectively). Item 9 (I hope the situation with PD improves) presented to DIF by PD duration and age, indicating that younger patients report higher coping scores than the older ones, for the same coping level. After splitting item 9 due to DIF by age, a good model fit was obtained, with χ^2^(18) = 33.471, *p* = 0.015, PSI = 0.765, unidimensionality, item local independency, item distribution M = 0.366, SD = 1.248, no DIF for the rest of the items, and a small ceiling effect (12.963%). Also, DIF by country in items 7 and 9 was cancelled with the top-down purification procedure (*p*-value = 0.160). The person-item distribution is shown in Fig. 1.

In the Integration dimension, item 18 (I have PD in mind when doing activities, tasks and/or plans) showed misfit (3.118) and therefore was removed, and the response scales of all items but one (item 20; Although I have PD I feel satisfied with my life) were recoded by collapsing the second and third response categories. The final model presented an adequate fit, χ^2^(16) = 28.23, *p* = 0.029, PSI = 0.728, unidimensionality, item local independency, no DIF by age, gender and HY stage. Item 17 presented DIF by PD duration; and items 18, 20 and 21 DIF by country, with an inconsistent pattern across items. The item distribution M = 0.238, SD = 1.203, and a small ceiling effect (9.878%; Fig. 1).

## Discussion

The LW-CI has been successfully used in PD studies in a wide range of countries [8] as well as in other chronic conditions [9]. However, this study makes an important contribution to understanding the measurement properties associated with the use of LW-CI in PD according to Rasch model, a powerful item response theory tool. This is the first study to analyze the psychometric properties of the LW-CI-PD using Rasch analysis.

The results emerged on this study indicate that the original LW-CI, when applied to PD patients, fits the Rasch model after performing some changes. The original scale included response categories that respondents are not totally able to differentiate, two subscales did not fit the model, three items that measured constructs other than living with PD, one redundant item was found, and the confirmation of multidimensionality was confirmed.

According to Rasch model, the LW-CI-PD is multidimensional, which supports the use of subscores instead of a single total score. This means that the LW-CI-PD measures different constructs (the subscales) and thus dimension scores should be used. This result completely fits with the theoretical framework carried out about living with a chronic illness as PD where it is defined as a multidimensional process with dynamical and cyclical characteristics [23]. According to theoretical bases and the results emerged in this study, the LW-CI-PD is a rating scale that will facilitate clinical specialists to identify the dimension(s) which is making a patient with PD develop negative and/or positive outcomes in the process of daily living with the disease. In this way, person-centred interventions could be developed accordingly, and consequently improving the patient’s quality of life and wellbeing.

Two subscales (Self-management and Adjustment) did not fit the Rasch model. This modification, pending of confirmation in further studies, might facilitate the usefulness of the LW-CI-PD in daily clinical practice since it provides a shorter and easier tool with direct applicability in patients with PD. More research is needed to confirm these results.

In the same way, four items were removed, one because of redundancy and two others because they measured constructs different from their respective dimensions. Item 8 (I try to lean on things that are important in my life) was removed because of redundancy. When applied to PD patients, trying to lean on things that are important in the patient life is probably more related to PD integration in daily living rather than to coping [24]. Items 2, 11 (I express my feelings so other people know how I feel and could help me) and 18 (I have PD in mind when doing activities, tasks and/or plans) were removed. Item 2 (I am ashamed of PD and I hide it to others) focuses on shame in PD, which might be personality-related and separate from acceptance [25, 26]. PD-related shame emerged from motor and non-motor symptoms, from loss of autonomy and need for help, and from perceived deterioration of body image [25], which is more associated with PD adaptation rather than acceptance. Item 11 (I express my feelings so other people know how I feel and could help me) was also removed confirming previous classic test analysis carried out in PD [8] showing inappropriate location according to the multitrait-scaling analysis and low inter-item correlation values. The content of item 18 (I have PD in mind when doing activities, tasks and/or plans) does not seem related to PD integration because having in mind PD is more connected to be aware of the disease and its symptoms, and in particular with non-motor symptoms [27, 28] rather than on integration. Besides, previous multitrait-item validity analysis carried out in PD [8] also corroborates the inappropriate location of item 18 in its domain (integration) and consequently, the suitability to remove it.

A unique contribution of Rasch analysis is the possibility of checking empirically how well the response categories are working. Several items showed disordered thresholds, which indicates that patients were not able to differentiate between the second and third response categories (rarely/almost nothing and sometimes/something). Indeed, the response categories “almost nothing” and “something” seem quite close. The score structure initially proposed for the LW-CI-PD [8] was done based on frequency distribution of raw scores. This study, pending confirmation by subsequent Rasch analyses, proposes the same response scale, but it requires different codification for the times. Instead of using a coding scheme from 0 to 4, items should be coded from 0 to 3 by giving the score 1 to the second and third response category.

All items were free from DIF by gender, age and disease severity, except one (item 9, I hope the situation with PD improves), which showed DIF by age. This indicates that older PD patients are more hopeless than younger PD patients. Younger patients overestimate scores, whereas older patients underestimate for the same coping level. PD population studies [29] did not find statistically significant results that could confirm this finding. However, in a general population, an association between older age and hopelessness was found [30]. To avoid the potential impact of this bias on the scale results, the item 9 was splitted with a good model fit. In addition, one Integration item showed bias by disease duration, and three Integration items and all Acceptance items displayed DIF by country. Caution should be taken when comparing results cross-nationally. However, further studies are needed to confirm these DIF results with larger samples and other settings.

According to the Rasch analysis, it is proposed a shorter modified version of the LW-CI-PD with 12 items grouped in three subscales and a simpler scoring scheme. However, further research is needed to replicate the results obtained in this study. Once replication of a stable model is achieved across different chronic diseases and countries, a conversion table can be used to transform raw scores to linear measures, without requiring a Rasch analysis for each data set.

This study presents some limitations relating the sample. The first concern is that only 1.54% of the sample presented advanced PD according to the H&Y staging (stage 5). This is common to many studies with PD patients and indicates the need to design studies targeted to advanced PD. It would be very interesting to perform Rasch analysis on a sample with advanced PD patients to examine how this would affect LW-CI-PD targeting. Another limitation of this study is that patients were recruited from different settings, forming a heterogeneous sample. However, this feature increases external validity.

## Conclusion

Through this Rasch analysis, unique information about the measurement properties of the LW-CI-PD has been provided. A shorter version, with fewer items and a simpler response scheme, is thus proposed. The resulting LW-CI-PD is a reliable, with good internal construct validity.

## Data Availability

The corresponding author had full access to all the data in the study and takes responsibility for the integrity and availability of the data and the accuracy of the data analysis.

## Abbreviations

PD: Parkinson’s disease
LW-CI-PD: Living with Chronic Illness Scale-PD
HY: Hoehn and Yahr
SCOPA-M: Scales for Outcomes in Parkinson’s Disease-Motor
CISI-PD: Clinical Impression of Severity Index-PD
